# Permanent tissue damage in multiple sclerosis lesions is associated with reduced pre-lesion myelin and axon volume fractions

**DOI:** 10.1177/13524585221110585

**Published:** 2022-07-28

**Authors:** Ian J Tagge, Ilana R Leppert, Dumitru Fetco, Jennifer SW Campbell, David A Rudko, Robert A Brown, Nikola Stikov, G Bruce Pike, Paul S Giacomini, Douglas L Arnold, Sridar Narayanan

**Affiliations:** McConnell Brain Imaging Center, Montreal Neurological Institute & Hospital, Montreal, QC, Canada; McConnell Brain Imaging Center, Montreal Neurological Institute & Hospital, Montreal, QC, Canada; McConnell Brain Imaging Center, Montreal Neurological Institute & Hospital, Montreal, QC, Canada; McConnell Brain Imaging Center, Montreal Neurological Institute & Hospital, Montreal, QC, Canada; McConnell Brain Imaging Center, Montreal Neurological Institute & Hospital, Montreal, QC, Canada; McConnell Brain Imaging Center, Montreal Neurological Institute & Hospital, Montreal, QC, Canada; Electrical Engineering, Polytechnique Montreal, Montreal, QC, Canada; Departments of Radiology and Clinical Neurosciences, Hotchkiss Brain Institute, University of Calgary, Calgary, AB, Canada; Neurology and Neurosurgery, Montreal Neurological Institute & Hospital, Montreal, QC, Canada; McConnell Brain Imaging Center, Montreal Neurological Institute & Hospital, Montreal, QC, Canada; McConnell Brain Imaging Center, Montreal Neurological Institute & Hospital, Montreal, QC, Canada

**Keywords:** MRI, multiple sclerosis, demyelination, quantitative MRI, biomarkers, T_2_ lesions

## Abstract

**Background:**

The use of advanced magnetic resonance imaging (MRI) techniques in MS research has led to new insights in lesion evolution and disease outcomes. It has not yet been determined if, or how, pre-lesional abnormalities in normal-appearing white matter (NAWM) relate to the long-term evolution of new lesions.

**Objective:**

To investigate the relationship between abnormalities in MRI measures of axonal and myelin volume fractions (AVF and MVF) in NAWM preceding development of black-hole (BH) and non-BH lesions in people with MS.

**Methods:**

We obtained magnetization transfer and diffusion MRI at 6-month intervals in patients with MS to estimate MVF and AVF during lesion evolution. Lesions were classified as either BH or non-BH on the final imaging visit using T_1_ maps.

**Results:**

Longitudinal data from 97 new T_2_ lesions from 9 participants were analyzed; 25 lesions in 8 participants were classified as BH 6–12 months after initial appearance. Pre-lesion MVF, AVF, and MVF/AVF were significantly lower, and T_1_ was significantly higher, in the lesions that later became BHs (*p* < 0.001) compared to those that did not. No significant pre-lesion abnormalities were found in non-BH lesions (*p* > 0.05).

**Conclusion:**

The present work demonstrated that pre-lesion abnormalities are associated with worse long-term lesion-level outcome.

## Background

The use of advanced magnetic resonance imaging (MRI) techniques, new imaging biomarkers, and high- and ultra-high field imaging in multiple sclerosis (MS) research has led to new insights in lesion evolution and disease outcomes. During the acute phase of lesion development, T_2_-weighted (-w) hyperintensity and gadolinium (Gd)-based contrast agent enhancement on T_1_-w MRI are both indicative of inflammation, with T_2_-w being sensitive to a variety of pathological processes leading to an increase in local water content, and Gd T_1_-w enhancement demonstrating blood–brain barrier compromise. Evolution from an acute enhancing lesion to a chronic T_1_-hypointense “black-hole” (BH) has long been used as evidence of irreversible tissue damage, the persistent T_1_-w hypointensity indicating loss of myelin and axons.^1,2^ Early in disease, some lesions are capable of repair and remyelination, and do not become BH lesions. The likelihood of lasting damage resulting in a BH has been shown to correlate with duration of Gd T_1_-w enhancement and volume of enhancing lesions,^1,3^ and recent work also suggests Gd T_1_-w enhancement pattern (i.e. ring-like vs nodular) and presence of a phase rim on susceptibility-weighted MRI are associated with long-term tissue damage in developing MS lesions.^4–6^

While T_1_-w and T_2_-w imaging are clinical standard of care and provide good sensitivity to disease activity, other MRI techniques are better suited to investigating specific pathological features such as demyelination or axonal loss. The magnetization transfer ratio (MTR) is a simple, semi-quantitative measure that is fast, commonly available, and sensitive to myelin content in vivo.^7,8^ Focal MTR decreases have been observed in normal-appearing white matter (NAWM) preceding appearance of Gd-enhancing or T_2_-w lesions in relapsing-remitting^9,10^ and progressive forms of MS,^10–12^ suggesting pre-lesion abnormalities may influence lesion formation. Diffusion-weighted MRI techniques offer insight into additional tissue properties that may reflect axonal integrity, and also are sensitive to pre-lesion abnormalities in MS NAWM.^13–16^ Combining myelin-sensitive metrics and diffusion MRI may give a more complete picture of the pathology.^17^

Understanding eventual lesion outcome is important for development of neuroprotective therapeutic strategies. NAWM is abnormal on MRI prior to lesion development, but it has not yet been determined if, or how, pre-lesional abnormalities relate to the long-term evolution of new lesions. To address this question, we obtained magnetization transfer (MT) and diffusion MRI at 6-month intervals in patients with MS to assess longitudinal changes during lesion evolution. Magnetization transfer saturation (MTSat) is a refined version of MTR that has a strong linear correlation with myelin content and allows estimation of myelin volume fraction (MVF).^18,19^ Neurite orientation dispersion and density imaging (NODDI) is a biophysical model of diffusion MRI that attempts to disentangle microstructural features obscured by traditional diffusion measures.^20^ Combined with estimates of MVF, NODDI can provide estimates of axonal volume fraction (AVF) that are less contaminated by lack of fiber direction coherence compared to traditional diffusion tensor imaging (DTI) parameters. We thus investigated the relationship between AVF and MVF abnormalities in NAWM preceding development of BH and non-BH lesions in people with MS.

## Methods

### Participants

Participants diagnosed with MS according to the 2010 McDonald Criteria^21^ were recruited from the MS clinic at the Montreal Neurological Institute-Hospital. Exclusion criteria included contraindications to MRI and/or Gd-based contrast agents. All subjects provided written informed consent in compliance with the local Research Ethics Board requirements.

### MRI acquisition

MRI data were collected at 6-month intervals (maximum four visits per subject) on a Siemens Prisma-Fit 3T instrument (Erlangen, Germany) equipped with a 64-channel phased-array head coil. A standardized MS protocol was acquired for T_2_-w, T_1_-w, new T_2_-w, and Gd-enhancing lesion segmentation: dual spin-echo PD/T_2_-w (repetition time (TR)/TE_1_ (echo time), TE_2_: 3000 ms/11, 99 ms; 1 mm × 1 mm × 2 mm voxel size; 72 axial slices); three-dimensional (3D) T_2_-w fluid-attenuated inversion recovery (FLAIR; TR/TE/inversion time (TI): 6000 ms/356 ms/2200 ms; 1 mm isotropic voxels; 176 sagittal slices); T_1_-w 3D gradient recalled echo (GRE) acquired before and after 0.1 mmol/kg Gd contrast (Gadovist, Bayer AG, Leverkusen, Germany) bolus injection (TR/TE/FA (flip angle): 28 ms/4.92 ms/25°; 1 mm isotropic voxels; 192 axial slices). Pre-contrast 3D MP2RAGE (TR/TE/FA_1_, FA_2_,/TI_1_, TI_2_: 5000 ms/2.76 ms/4°, 5°/940 ms, 2830 ms; 1 mm isotropic voxels; 208 sagittal slices) provided quantitative T_1_ maps for BH lesion classification.

The diffusion protocol consisted of one signal average at *b*-values of 300 s/mm^2^ (10 directions), 700 s/mm^2^ (30 directions) and 2500 s/mm^2^ (64 directions) and nine *b* = 0 s/mm^2^ images, acquired using a single-shot, spin-echo echo-planar imaging (EPI) sequence^20^ with monopolar gradients (TR/TE: 3000 ms/65 ms; 2 mm isotropic voxels; GRAPPA factor 2 and multiband^22^ acceleration factor of 3). Magnetic susceptibility–induced distortions were corrected using a blip-up, blip-down phase-encode strategy.^23^

The MTSat protocol was based on the work of Helms et al.,^18,24^ using a set of non-selective 3D FLASH (fast low angle shot) acquisitions to obtain MT-weighted (TR/TE/FA: 36 ms/4.92 ms/5°; MT preparation pulse: FA 500°, offset frequency +1200 Hz, pulse width 9.984 ms; 1 mm isotropic voxels; 192 axial slices), PD-w (FA 5°; no MT pulse) and T_1_-w (TR/FA: 28 ms/25°; no MT pulse) images. A pair of low spatial resolution–segmented EPI acquisitions (TR/TE: 4010 ms/46 ms; FA 1/FA 2: 60°/120°; 2 mm × 2 mm × 4 mm voxel size; 35 axial slices) were acquired to calculate B_1_^+^.

### Image processing

Most image processing was done using locally developed tools; all images from all timepoints were registered into a patient-specific common reference space for analysis, as previously described.^25^ T_2_-w lesions were segmented on baseline data using a locally developed, automated, multispectral Bayesian technique^26^ using the PD-w, T_2_-w, FLAIR and T_1_-w images. Resulting lesion labels were reviewed and corrected as necessary by a trained reader using interactive software, as previously described.^27^ New T_2_-w lesions arising from previously NAWM were similarly identified on follow-up data and then manually corrected by an expert reviewer. New T_2_-w lesions were further manually classified as either (1) de novo new T_2_-w, arising entirely from NAWM with no adjacent voxels labeled as previously existing T_2_-w lesions or (2) enlarging/expanding new T_2_, which are areas of new T_2_-w hyperintensity adjoining T_2_-w lesions identified on the previous timepoint. Chronic T_1_-w BH regions within T_2_-w lesions were segmented automatically on quantitative T_1_ map reconstructions from the pre-contrast MP2RAGE acquisition at the final imaging timepoint to avoid transient T_1_ changes associated with acute inflammation during lesion formation. T_2_-w lesions containing clusters of at least eight spatially connected voxels with T_1_ > 1600 ms at the final timepoint were classified as BH-fate lesions. Gd-enhancing lesions were segmented on post-contrast T_1_-w images with reference to the registered pre-contrast T_1_-w images.

**Figure 1.**
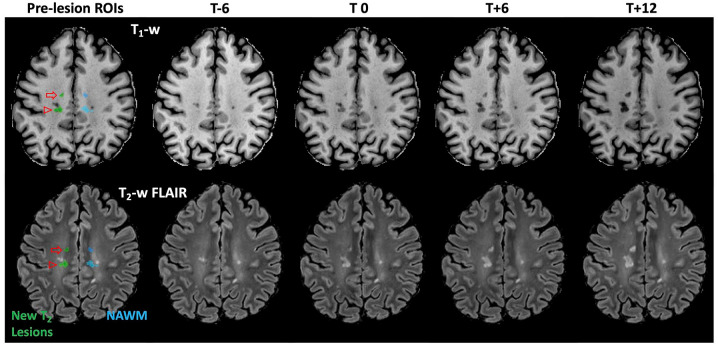
Representative lesion masks. New T_2_-w lesion masks (green) and spatially matched contralateral NAWM masks (blue) are overlaid on the pre-lesion (6 months prior to lesion appearance (T-6)) T_1_-w anatomical (top) and T_2_-w FLAIR (bottom) images. The anterior lesion (open arrow) is a discrete de novo lesion arising entirely from NAWM with no voxels spatially connected to existing lesions. The second lesion (open arrowhead) is an expanding/enlarging lesion, and the spatially matched contralateral NAWM ROI was manually adjusted to avoid an existing T_2_ lesion while maintaining total ROI volume.

Myelin and axonal density vary spatially throughout the brain, so spatially matched contralateral NAWM regions of interest (ROIs) in homologous areas of the opposite hemisphere were used for pairwise analyses.^9,10^ Contralateral NAWM ROIs were prepared by first creating cumulative non-WM masks for each participant by summing tissue classification masks from all timepoints for gray matter, T_2_-w lesions, and cerebrospinal fluid, then dilating 1 mm to reduce partial volume contamination. Lesion masks were mirrored across the midline and non-WM masks were applied to ensure each ROI resided entirely within the NAWM at every imaging timepoint. ROIs were reviewed and manually corrected to ensure contralateral NAWM ROI volumes were matched to the corresponding lesion while allowing for anatomical variation between hemispheres. Representative ROI pairs are illustrated in Figure 1.

**Figure 2.**
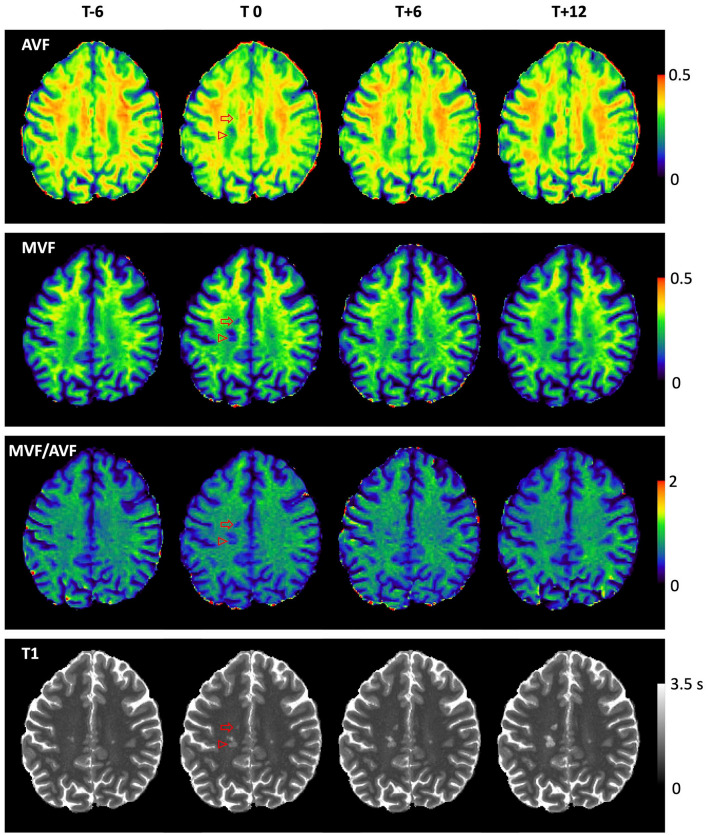
Parametric maps showing lesion evolution. Parametric maps demonstrating lesion evolution in the de novo (open arrow) and expanding/enlarging (open arrowhead) lesions from Figure 1. AVF change is not visually appreciable in this slice at T_0_ in the de novo lesion, where axonal loss follows acute inflammation and demyelination. AVF, MVF, and T_1_ worsen progressively over time in both lesions, ultimately resulting in black-hole fate at T+12.

MTSat maps were computed with B_1_^+^-correction for non-uniform radio-frequency (RF) transmit field effects as previously described^18,28–30^ using pipelines developed locally using the Montreal Neurological Institute (MINC) toolkit (http://bic-mni.github.io/). MVF estimates were obtained from MTSat using a calibration factor obtained from a combined MRI/histology data set with the assumption of a linear relationship and zero-intercept.^31^ NODDI processing was performed using the freely available “AMICO” implementation of the NODDI model^32^ to obtain estimates of AVF

(1) $$\mathrm{AVF}=\left(1-\mathrm{MVF}\right)\left(1-f_{iso}\right)f_{in}$$

where *f_iso_* and *f_in_* are the isotropic and restricted (intra-neurite) signal fractions, respectively, also obtained from the NODDI model. While MVF provides an informative estimate of myelin content within a voxel, interpretation of changes in MVF will be different in a lesion with minor axonal loss versus a lesion with more profound axonal loss. For example, limited recovery of MVF may reflect poor remyelination if axons are spared but could indicate relatively good remyelination in the context of substantial axonal loss, where overall (stable) change in MVF (i.e. MVF_drop_ = MVF_post_ − MVF_pre_) is determined to some extent by the number of remaining axons available to remyelinate. To investigate relative remyelination within lesions while controlling for the number of axons within a voxel, we use the ratio MVF/AVF as a normalized MVF metric.^31^ Parametric maps demonstrating lesion evolution are shown in Figure 2.

### Statistical analysis

**Figure 3.**
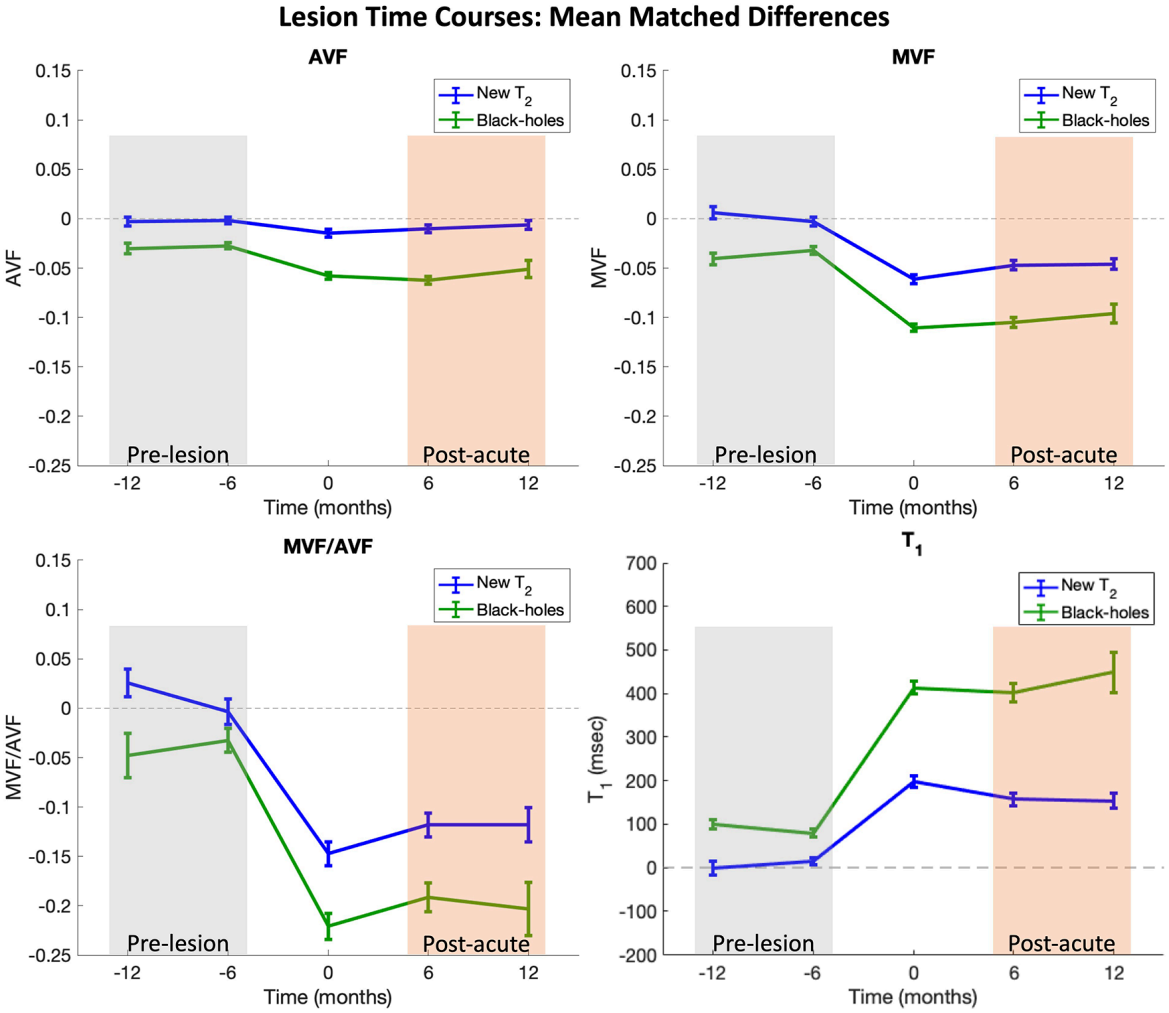
Time course plots. Time course plots represent mean differences between lesion ROIs and matched contralateral NAWM ROIs. Contralateral ROIs were masked to remove non-WM tissue (i.e. cortex, lesion, cerebrospinal fluid) and were subsequently manually adjusted to maintain consistent ROI volume. Time courses were shifted in time such that Time 0 corresponds to the first time each lesion was observed on MRI. Lesions containing clusters of at least 8 connected voxels with T_1_ > 1600 ms at the last imaging timepoint were classified as black-hole lesions (plotted in green). Bars represent the standard errors. Pre-lesion and post-acute values are calculated for each lesion by averaging all available timepoints in the gray- and orange-shaded boxes, respectively.

Pre-lesion and post-acute values were obtained for each ROI by averaging all available timepoints before, or after, lesion appearance, corresponding to the gray- and orange-shaded areas in Figure 3, respectively. Longitudinal changes within ROIs were calculated by subtracting post-acute ROI averages from pre-lesion ROI averages. One-sided paired *t*-tests were used to compare pre-lesion and post-acute values within ROIs. BH and non-BH lesion metrics, including ROI volumes, were compared with mixed-effect models with primary outcome (e.g. MVF_drop_) as fixed effect and subject as random effect. Linear mixed-effects models were also used to compare BH and non-BH lesion to contralateral NAWM with ROI tissue type (i.e. NAWM, BH, non-BH) as fixed effect and lesion ID (i.e. a lesion and its corresponding contralateral NAWM ROI would have a common ID such as subject-X_lesion-Y) as a random effect; significance was set at the 0.05 level for all comparisons. Associations between lesion type (discrete/de novo or enlarging/expanding) and lesion fate were evaluated with a chi-square test.

## Results

A total of 40 people with MS were recruited for this study: 29 with relapsing-remitting multiple sclerosis (RRMS), 10 with secondary-progressive multiple sclerosis (SPMS), and 1 with primary-progressive multiple sclerosis (PPMS). One participant classified as having RRMS was subsequently diagnosed with myelin oligodendrocyte glycoprotein antibody disease (MOGAD), and was removed from analysis. Ten participants withdrew (four after baseline visit, one after completing two visits, six after completing three visits); seventeen participants were lost to follow-up due to the COVID-19 pandemic (nine after baseline, three after completing two visits, five after completing three visits). Longitudinal data were available from twenty-five participants (thirteen with four completed visits, nine with three completed visits, three with two completed visits); demographics for these 25 participants are presented in Table 1. New T_2_ lesions (*n* = 118) were identified in 13 participants (10 RRMS, 3 SPMS). New T_2_ lesions were found only at the final timepoint in one participant, and three other participants with new T_2_ lesions were lost to follow-up due to the COVID-19 pandemic. Summary demographics comparing the participants with and without observed new lesions are given in Table 2. Briefly, the group with new lesions was slightly, but not significantly (unpaired *t*-test *p* > 0.05), younger with shorter disease duration, lower baseline Expanded Disability Status Scale (EDSS), and more EDSS increase during observation period, and had significantly higher annualized relapse rate (*p* = 0.03) compared to subjects with no new lesions. Finally, pre-lesion and post-acute data were available for 97 lesions from 9 participants (7 RRMS, 2 SPMS). Six of the nine subjects included in the longitudinal analysis were on DMT (three Teriflunomide, two Copaxone, one Gilenya). One SPMS subject (on Copaxone) did not have new BH on study. Twenty-five lesions in eight participants met our criteria for T_1_ BHs 6–12 months after lesion appearance. Eight lesions were Gd-enhancing at first observation, five of which had BH fate. Lesions with BH fate had significantly larger volume at first appearance than non-BH lesions (mean volume (±standard deviation): 257.8 (±243.9) vs 66.4 (±74.3) mm^3^; *p* < 0.001) but were not significantly more likely to arise from either enlarging/expanding or discrete/de novo lesions (chi-square *p* = 0.09).

**Table 1.** Baseline demographic characteristics.

Subtype	M/F	Age (years)^a^	Disease duration^a^	EDSS^b^	On DMT
RRMS	5/13	47.8 (±10.7)	16.3 (±8.5)	2	14^c^
SPMS	1/5	57.5 (±7.3)	22.5 (±7.8)	4.5	3^d^
PPMS	0/1	66 (±0)	38 (±0)	6.5	0

RRMS: relapsing-remitting multiple sclerosis; SPMS: secondary-progressive multiple sclerosis; PPMS: primary-progressive multiple sclerosis; EDSS: Expanded Disability Status Scale.

a Mean value (±standard deviation).

b Median.

c Alemtuzumab: *n* = 1, Avonex: *n* = 2, Copaxone: *n* = 5, Gilenya: *n* = 1, Teriflunomide: *n* = 5.

d Copaxone: *n* = 1, Ocrelizumab: *n* = 1, Teriflunomide: *n* = 1.

**Table 2.** Baseline demographics comparing participants with and without new T_2_ lesions.

	M/F	Age (years)	Baseline EDSS	EDSS change	Disease duration (years)	Number of MRI visits	Number of relapses	ARR	Participants with relapses
Participants with new lesions	5/8	47.62 (±12.5)	2.5 (±1.93)	0.5 (±0.98)	15.69 (±8.14)	3.31 (±0.63)	0.46 (±0.66)	0.3 (±0.52)	5
Participants without new lesions	1/11	54.33 (±8.09)	3.25 (±1.73)	0.0 (±0.39)	21.83 (±9.81)	3.33 (±0.78)	0.08 (±0.29)	0.06 (±0.19)	1
*p*-value		0.06	0.31	0.07	0.05	0.46	0.04	0.03	

EDSS: Expanded Disability Status Scale; MRI: magnetic resonance imaging; ARR: annualized relapse rate.

Values reported as mean value (±standard deviation); EDSS and EDSS change are reported as median (±standard deviation).

*p*-values determined by unpaired one-tailed *t*-tests.

### Backward analysis of pre-lesion abnormalities

**Figure 4.**
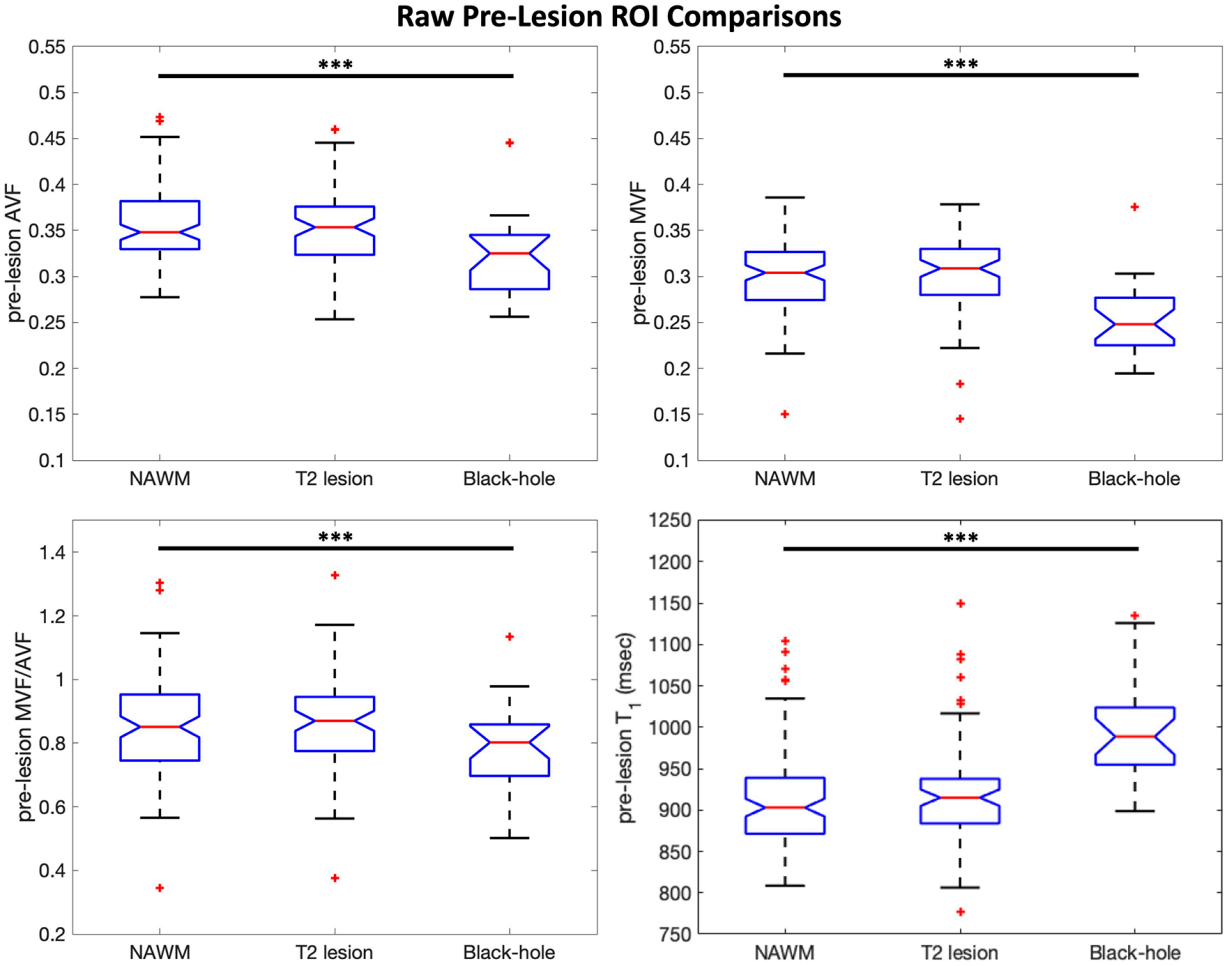
Pre-lesion boxplots. Boxplots comparing pre-lesion values (calculated for each ROI by averaging all available timepoints in the Figure 3 gray-shaded boxes) between lesion ROIs and contralateral NAWM ROIs matched in size and homologous location to each lesion ROI. Pre-lesion MVF, AVF, MVF/AVF, and T_1_ in black-hole lesions are significantly different from contralateral NAWM. Mixed-effects models were used to calculate *p*-values and significance was set at the 0.05 level. Outliers are indicated by red + symbols. ***p < 0.001.

ROI time courses for mean matched differences (e.g. ≡ MVF_lesion_ − MVF_NAWM_) are shown in Figure 3. Time courses were temporally shifted such that Time 0 corresponds to the first time each lesion was observed on MRI. In these plots, values close to 0 on the *y*-axis indicate no difference between lesion and contralateral NAWM; qualitatively, non-BH lesions were very similar to matched contralateral NAWM prior to T_2_ lesion appearance (gray shading) and areas where BH lesions formed were abnormal compared to contralateral NAWM before T_2_ lesion appearance. The largest deviations away from 0 are observed at lesion appearance where acute inflammation is likely captured in addition to frank tissue damage. Some recovery is then observed in the post-acute period (orange shading) for both BH and non-BH lesions. Boxplots in Figure 4 compare raw pre-lesion AVF, MVF, MVF/AVF, and T_1_ values for each ROI group. The pre-lesion MVF, AVF, and MVF/AVF were significantly lower, and T_1_ was significantly higher, in the ROIs that later became BHs (mixed-effects models, *p* < 0.001) compared to those that did not. No significant pre-lesion abnormalities were found in non-BH ROIs (*p* > 0.05).

### Forward analysis of residual post-acute abnormalities relative to NAWM

**Figure 5.**
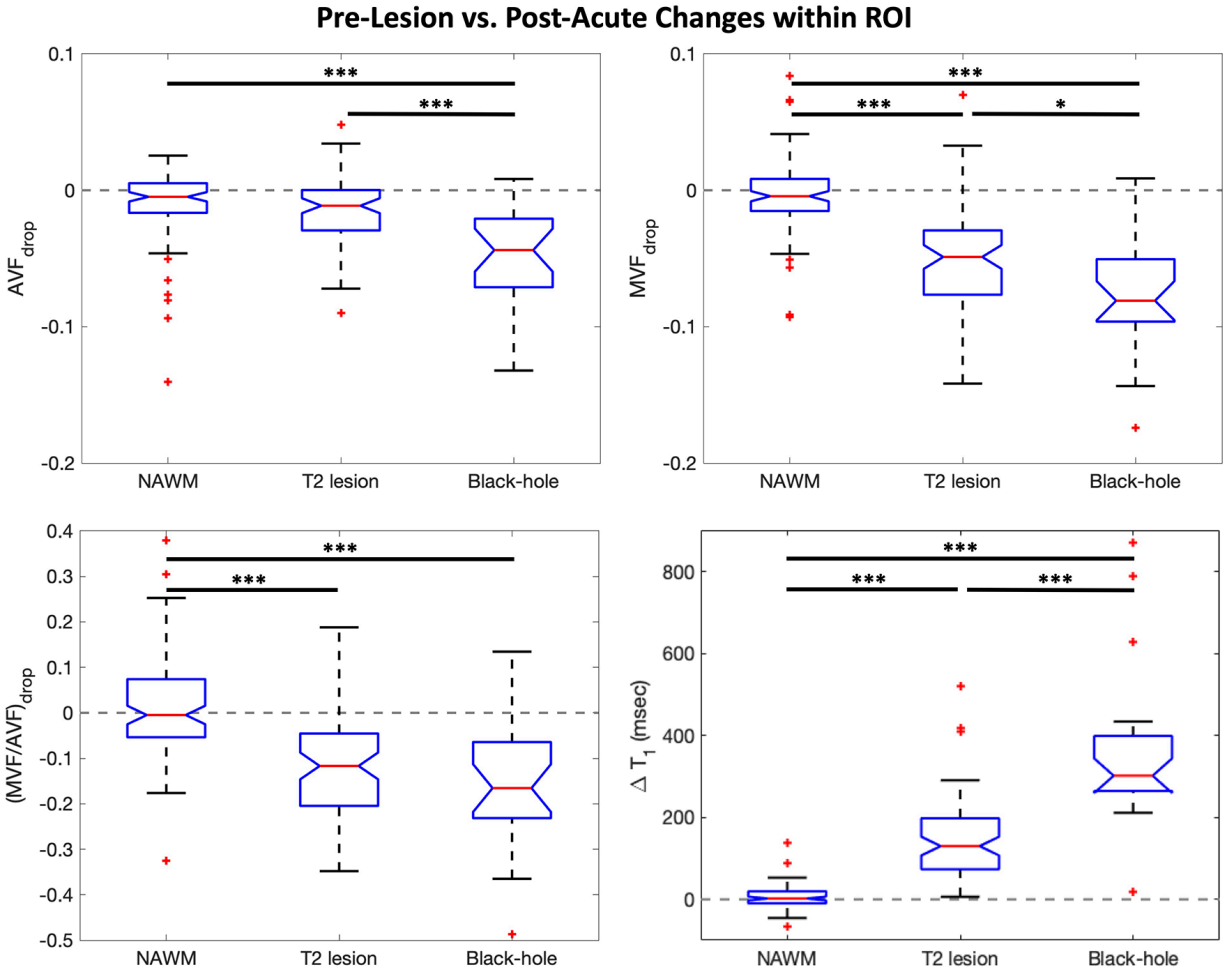
Parameter change boxplots. Boxplots illustrating parameter change for each lesion type and NAWM (i.e. MVF_drop_ = post-acute MVF − pre-lesion MVF). On average, NAWM ROIs showed no significant change over time. Aside from AVF in non-BH lesions, all other signals showed longitudinal changes that were significantly greater than in contralateral NAWM ROIs matched in size and homologous location to each lesion ROI. Recovery in BH lesions was substantially attenuated compared to non-BH lesions: MVF and AVF were significantly decreased, and T_1_ was significantly increased (*p* < 0.001 for all comparisons). Interestingly, (MVF/AVF)_drop_ was not different between BH and non-BH lesions. Mixed-effects models were used to calculate *p*-values and significance was set at the 0.05 level. Outliers are indicated by red + symbols. *p < 0.05; ***p < 0.001.

Post-acute lesions demonstrated significant abnormalities compared to contralateral NAWM, and BH lesions demonstrated greater residual tissue damage (i.e. greater initial acute injury and/or poorer recovery) compared to non-BH lesions. All markers were significantly decreased in post-acute BH compared to NAWM (AVF in non-BH, *p* = 0.03, all others *p* < 0.001). We estimated the magnitude of change in each parameter from a pre-lesion timepoint to a stable post-acute timepoint (>3 months after lesion appearance), as has been previously reported in normalized MTR.^33^ This approach provides a measure of unrepaired tissue injury associated with the new lesion, while avoiding the acute phase when inflammation and edema are most likely to affect MRI metrics, and is not sensitive to the precise timing of the MRI scans with respect to the time of lesion formation. Boxplots in Figure 5 show parameter changes between pre-lesion and post-acute measurements; hence, 0 represents no net change over time within each ROI. On average, NAWM showed no change between pre-lesion and post-acute measurements.

### Within-lesion changes as a measure of unrepaired tissue injury

While AVF_drop_ was qualitatively, but not significantly (*p* = 0.08), different from NAWM in non-BH lesions, both BH and non-BH lesions showed changes in MVF, MVF/AVF, and T_1_ that were significantly greater (*p* < 0.001) than in spatially matched contralateral NAWM. AVF_drop_ was significantly different from spatially matched contralateral NAWM only in BH lesions. Mixed-effects models excluding NAWM ROIs demonstrated additional significant differences between BH and non-BH lesions. Axon and myelin loss, estimated by AVF_drop_ and MVF_drop_, respectively, were significantly greater in BH lesions compared to non-BH lesions (*p* < 0.001 for all comparisons). T_1_ increase was significantly greater in BH than non-BH lesions (*p* < 0.001). However, change in relative myelination, estimated as (MVF/AVF)_drop_, was not different between the two lesion types.

## Discussion and conclusion

Longitudinal evaluation of MRI microstructural markers provides important insight into disease pathogenesis and lesion evolution in MS. Previous studies provided evidence of NAWM abnormalities preceding lesion formation, and we demonstrate here that the magnitude of pre-lesion abnormalities is associated with lesion outcomes. We show that MVF and AVF are uniquely decreased (compared to spatially matched contralateral NAWM) preceding appearance of new T_2_ lesions that eventually become BHs, suggesting subtle pathology beginning at least several months before T_2_ lesion appearance is associated with more severe tissue injury and loss.

The present work demonstrated that pre-lesion abnormalities are associated with worse long-term lesion-level outcome, extending prior observations of pre-lesion abnormalities.^9–15^ Large pre-lesion abnormalities observed in this study could reflect previous pathological insult that was (1) subclinical: did not create clinical event, was not captured on MRI, and resolved sufficiently to not meet T_2_ lesion criteria or (2) subacute: pathological event associated with low-grade, focal inflammation below detection threshold for what would be considered T_2_ lesion. In either case, such regions represent tissue affected by microstructural changes and/or inflammation^34^ that would be susceptible to greater damage after a second or new hit. Alternatively, it could reflect intrinsic characteristics of tissue that is more likely to develop more severe lesions.

The AVF_drop_ and MVF_drop_ results are intuitive and consistent with what is known about BH and non-BH lesions: BH lesions are associated with substantial axonal loss, which is reflected in significantly decreased AVF; non-BH lesions exhibit demyelination with relatively preserved axonal content, consistent with small and non-significant AVF_drop_. Both lesion types show substantial demyelination which is reflected as significant MVF_drop_. The magnitude of change is higher in MVF than in AVF for both BH and non-BH lesions, suggesting primary tissue damage is demyelination in both lesion types.

We found preliminary evidence of no difference in relative myelin content change (MVF/AVF)_drop_ between BH and non-BH lesions. This finding suggests that, at the level of the axon, the extent of remyelination of surviving axons was similar between BH and non-BH lesions. Thus, because the extent of remyelination per axon is similar between the two lesion types, the larger drops in BH lesions are likely due to greater axonal loss during acute lesion formation.^35^ At the aggregate level, this results in lower AVF and MVF (since all the myelin associated with degenerated axons is also lost).

### Limitations

This methodology used for MVF and AVF estimation from MRI data is at the development stage, and relies on several assumptions that have been detailed elsewhere.^19,36^ The importance and influence of assumptions such as equal intra- and extra-neurite T_2_ relaxation times^37^ and fixed diffusivities in the NODDI model are unclear and need further investigation in the context of MS pathology. For example, if the extra-axonal T_2_ is elevated as has been reported in age-related white matter lesions,^38^ the MVF/AVF differences between non-BH and BH lesions could be underestimated.

Due to participant withdrawal and restricted in-person research during the COVID-19 pandemic, some lesions only have ~6 months follow-up. However, previous work has demonstrated that MTR in Gd-enhancing lesions typically is fully resolved by 6 months;^39^ it is reasonable to assume all BH lesions were at least 6 months old at the final visit, and thus transient edema and other acute effects that may spuriously increase T_1_ should be resolved and not result in false-positive BH classification.

This study was not powered to evaluate associations between lesion outcomes and clinical outcomes. However, recently BH lesions have been used primarily to evaluate tissue damage and demonstrate protective effects of DMTs rather than to predict clinical course.^40,41^ Larger studies enrolling more subjects with longer follow-up periods will be necessary to investigate the association between pre-lesion abnormalities and conversion to chronic active/slowly expanding lesions, to assess associations with clinical outcomes such as recovery (or not) from relapses, and to establish whether pre-lesion abnormalities can be used to build a model predictive of lesion fate that can be used to determine outcomes in trials of neuroprotective/remyelination therapies.

### Summary

We report two important novel observations regarding MS lesion evolution: (1) that more destructive BH lesions arise from tissue with more severe pre-lesional abnormalities and (2) that more destructive BH lesions may have an MVF/AVF that is the same as less destructive lesions but less than NAWM, suggesting that there may be room for improved remyelination independent of lesion severity.
